# Evidence for cryptic molting behavior in the trilobite *Toxochasmops vormsiensis* from the Upper Ordovician Katian Kõrgessaare Formation, Estonia

**DOI:** 10.1007/s00114-024-01906-8

**Published:** 2024-04-12

**Authors:** Russell D. C. Bicknell, Ernesto E. Vargas-Parra, Neil H. Landman, Helje Pärnaste

**Affiliations:** 1 Division of Paleontology (Invertebrates), American Museum of Natural History, New York, NY 10024 USA; 2 Palaeoscience Research Centre, School of Environmental & Rural Science, University of New England, Armidale, NSW 2351 Australia; 3 Institute of Geology at Tallinn University of Technology, 19086 Tallinn, Estonia

**Keywords:** Cryptic behavior, Molting, Trilobite, Ordovician, Nautiloid, *Gorbyoceras*, Paleoecology

## Abstract

Documentation of cryptic trilobite behavior has presented important insights into the paleoecology of this fully extinct arthropod group. One such example is the preservation of trilobites inside the remains of larger animals. To date, evidence for trilobites within cephalopods, gastropods, hyoliths, and other trilobites has been presented. Importantly, most of these interactions show trilobite molts, suggesting that trilobites used larger animals for protection during molting. To expand the record of molted trilobites within cephalopods, we present a unique case of a *Toxochasmops vormsiensis* trilobite within the body chamber of a *Gorbyoceras textumaraneum* nautiloid from the Upper Ordovician Kõrgessaare Formation of Estonia. By examining this material, we present new insights into the ecology of pterygometopid trilobites, highlighting how these forms used large cephalopods as areas to successfully molt.

**Supplementary information:**

The online version contains supplementary material available at 10.1007/s00114-024-01906-8.

## Introduction

Examination of trilobite molting patterns and processes has presented insight into the paleoecology of these extinct arthropods (Henningsmoen 1975; McNamara and Rudkin 1984; Speyer 1985; Daley and Drage 2016; Drage and Daley 2016; Drage et al. 2018; Drage 2019, 2024). The exceptional preservational potential of trilobite exoskeletons permitted this line of inquiry (Whittington 1990; Daley and Drage 2016), resulting in a comprehensive understanding of molting configurations and long-term evolutionary patterns (Daley and Drage 2016; Drage and Daley 2016; Drage et al. 2018, 2023; Drage 2019, 2024). As such, trilobite molting processes are known across most of the Paleozoic and among higher order groupings.

Various forms of cryptic trilobite behavior have been documented, including clustering, hiding, and gregariousness (Brett 1977; Davis et al. 2001; Paterson et al. 2008; Popp and Pärnaste 2011; Fatka and Budil 2014; Bicknell et al. 2019; Fatka et al. 2021; Bicknell and Kimmig 2023). Additionally, there are rare records of smaller trilobites preserved within the remains of larger animals, such as cephalopods, other trilobites, and brachiopods (Table 1; Brett 1977; Valent et al. 2008; Fatka et al. 2009, 2021; Fatka and Szabad 2011; Fatka and Kozak 2014). This so-called conchicolous habit (= “the use by other animals of shells as residences after the original builders have died” Vermeij 1987, p. 240), or inquilinism (sensu Fraaye and Jäger 1995b; Landman et al. 2014; Fraaije et al. 2020; Bicknell et al. 2021), has been documented in 15 trilobite genera spanning the Cambrian through the Carboniferous (Table 1; Fatka and Kozak 2014; Fatka et al. 2021). These associations have been attributed to feeding on carcasses (Fatka et al. 2021), habitation and/or shelter (Rakociński 2009; Vokáč et al. 2015; Fatka et al. 2021), or using larger animals for protection during molting (Ladd 1929; Chatterton et al. 2003; Fatka et al. 2008, 2021; Zong et al. 2016). To expand the record of molted trilobites preserved within cephalopods, we present a molted *Toxochasmops vormsiensis* Rõõmusoks 1998 within the body chamber of a nautiloid cephalopod (*Gorbyoceras textumaraneum* (Roemer 1861), identified by Björn Kröger pers. comms.) from the Upper Ordovician Kõrgessaare Formation, Vormsi, Estonia. This record presents the first example of cryptic molting for pterygometopid trilobites.

**Table 1:** Summary of trilobites within host groups. Ordered temporally and then taxonomically by genus. Note that there has been additional documentation of trilobites within larger animals (see Mikulic 1994; Feist 2001). However, these lacked geological information so could not be included within this table. The symbol “–” indicates that the data was not presented in the manuscript

Trilobite species	Trilobite order	Trilobite family	Host	Host species	Age	Formation	Explanation	Citation
Cambrian								
*Skreiaspis spinosa* (Pompeckj 1895)	Redlichiida	Agraulidae	Hyolith	*Maxilites maximus* (Barrande 1867)	Drumian	Buchava Formation, Czech Republic	Refuge for molting	Fatka et al. (2008, pl. 1, Fig. 1), Valent et al. (2008, Fig. 1)
*Skreiaspis spinosa*	Redlichiida	Agraulidae	Trilobite, Ptychopariidae	*Ptychoparia dubinka* Kordule 2006	?Drumian	?Buchava Formation, Czech Republic	Hiding	Fatka et al. (2008, pl. 1, Fig. 6)
Ordovician								
*Eoharpes* sp.	Harpida	Harpetidae	Indeterminate cephalopod	–	Middle Ordovician (Darriwilian)	Šárka Formation, Czech Republic	Molting	Fatka et al. (2021, Fig. 3E)
*Eoharpes* sp.	Harpida	Harpetidae	Illaenid trilobite	*Ectillaenus katzeri katzeri* (Barrande 1872)	Middle Ordovician (Darriwilian)	?Šárka Formation, Czech Republic	Molting	Fatka et al. (2021, Fig. 4A)
*Eoharpes primus* (Barrande 1872)	Harpida	Harpetidae	Asaphid or illaenid trilobite	–	Middle Ordovician (Darriwilian)	Šárka Formation, Czech Republic	Feeding or hiding	Fatka et al. (2021, Fig. 4B)
*Eoharpes primus*	Harpida	Harpetidae	Illaenid trilobite	*Ectillaenus* sp.	Middle Ordovician (Darriwilian)	Šárka Formation, Czech Republic	Feeding or hiding	Fatka et al. (2021, Fig. 4D)
*Eoharpes primus*	Harpida	Harpetidae	Indeterminate trilobite	–	Middle Ordovician (Darriwilian)	Šárka Formation, Czech Republic	Shelter	Fatka et al. (2021, Fig. 4E)
*Placoparia* sp.	Phacopida	Pliomeridae	Indeterminate pseudorthocerid cephalopod	–	Middle Ordovician (Darriwilian)	Šárka Formation, Czech Republic	Carcasses and possible feeding	Fatka et al. (2021, Fig. 3B1, B2)
*Placoparia* sp.	Phacopida	Pliomeridae	Actinocerid cephalopod	“*Orthoceras*” cf. *bonum* Barrande 1867	Middle Ordovician (Darriwilian)	Šárka Formation, Czech Republic	Feeding or hiding	Fatka et al. (2021, Fig. 3C1, C2)
*Placoparia* sp.	Phacopida	Pliomeridae	Indeterminate orthoconic cephalopod	–	Middle Ordovician (Darriwilian)	Šárka Formation, Czech Republic	Molting	Fatka et al. (2021, Fig. 3D)
*Placoparia* sp.	Phacopida	Pliomeridae	Cyclopygid trilobite	*Degamella princeps* (Barrande 1872)	Middle Ordovician (Darriwilian)	Šárka Formation, Czech Republic	Molting	Vokáč et al. (2015, pl. 2, Figs. 1–5), Fatka et al. (2021, Fig. 4F1–F3)
*Placoparia cambriensis* Hicks 1875	Phacopida	Pliomeridae	Asaphid trilobite	*Ogyginus forteyi* Adrain & Westrop 2005	Middle Ordovician (Darriwilian)	Valongo Formation, Portugal	Possible molting	Gutiérrez-Marco et al. (2009, Fig. 4C)
*Placoparia (Placoparia) cambriensis* Hicks 1875	Phacopida	Pliomeridae	Indeterminate endoceratid cephalopod	–	Middle Ordovician (Darriwilian)	Šárka Formation, Czech Republic	Possible feeding	Fatka et al. (2021, Fig. 3A)
*Placoparia (P.) cambriensis*	Phacopida	Pliomeridae	Dalmanitid trilobite	*Ormathops atavus* (Barrande 1872)	Middle Ordovician (Darriwilian)	Šárka Formation, Czech Republic	Feeding or hiding	Vokác et al. (2019, pl. 3, figs. 4, 5), Fatka et al. (2021, Fig. 3G)
*Placoparia* (*P*.) *cambriensis*	Phacopida	Pliomeridae	Indeterminate cephalopod	–	Middle Ordovician (Darriwilian)	Šárka Formation, Czech Republic	Feeding or hiding	Vokáč et al. (2015, pl. 3, Fig. 6), Fatka et al. (2021, Fig. 3H)
*Placoparia* (*P*.) *cambriensis*	Phacopida	Pliomeridae	Asaphid trilobite	*Asaphellus desideratus* (Barrande 1872)	Middle Ordovician (Darriwilian)	Šárka Formation, Czech Republic	Feeding	Vokáč et al. (2015, pl. 3, Fig. 6), Fatka et al. (2021, Fig. 4C)
*Placoparia* (*P*.) *cambriensis*	Phacopida	Pliomeridae	Asaphid trilobite	*Asaphellus desideratus*	Middle Ordovician (Darriwilian)	–	Feeding or hiding	Fatka et al. (2021, Fig. 3G1, G2)
*Eoharpes benignensis* (Barrande 1872)	Harpida	Harpetidae	Asaphid trilobite	*Nobiliasaphus repulses* Přibyl & Vaněk, 1965	Middle-Upper Ordovician (Darriwilian-Sandbian)	Dobrotivá Formation, Czech Republic	Reproduction, molting, hiding, or food	Fatka and Budil (2014, Figs. 4, 5)
*Eoharpes primus*	Harpida	Harpetidae	Indermanent illaenid or asaphid trilobite	–	Middle-Upper Ordovician (Darriwilian-Sandbian)	Dobrotivá Formation, Czech Republic	Feeding or hiding	Fatka et al. (2021, Fig. 3F1, F2)
*Eoharpes cristatus* Romano 1975	Harpida	Harpetidae	Indeterminate nautiloid	–	Upper Ordovician (Sandbian)	Queixopêrra Member, Cabeço do Peáo Formation, Portugal	Protection or molting	Pereira et al. (2015)
*Isotelus gigas* DeKay 1824	Asaphida	Asaphidae	Indeterminate cephalopod	–	Upper Ordovician (Sandbian- Katian)	Platteville Formation, Iowa, USA	–	Davis et al. (2001, Fig. 6)
*Acidaspis* sp.	Odontopleurida	Odontopleuridae	Proteoceratid cephalopod	*Treptoceras* sp.	Upper Ordovician (Katian)	–	Molting	Davis et al. (2001, Fig. 5)
*Anataphrus vigilans* (Meek and Worthen 1875)	Asaphida	Asaphidae	Indeterminate cephalopod	–	Upper Ordovician (Katian)	Maquoketa Formation, Iowa, USA	Retreat or molting	Ladd (1929, p. 387)
*Flexicalymene meeki* (Foerste 1910)	Phacopida	Calymenidae	Platyceratid gastropod	*Cyclonema* sp.	Upper Ordovician (Katian)	?Waynesville Formation, Cincinnati, Ohio, USA	Molting	Brandt (1993, Fig. 3.6)
*Flexicalymene meeki*	Phacopida	Calymenidae	Nautiloid cephalopod	?*Treptoceras duseri* (Hall and Whitfield 1875)	Upper Ordovician (Katian)	–	Molting	Davis et al. (2001, Figs. 2–4)
*Toxochasmops vormsiensis*	Phacopida	Pterygometopidae	Nautiloid cephalopod	*Gorbyoceras textumaraneum*	Upper Ordovician (Katian)	Kõrgessaare Formation, Estonia	Molting	This paper, Fig. 2
Silurian								
*Harpes* cf*. acuminatus* Lindström, 1885	Harpida	Harpetidae	Indeterminate nautiloid cephalopod	–	Ludlow (Gorstian)	Hemse Beds, Gotland, Sweden	Different molting events of same trilobite	Zwanzig and Liebermann (2012)
*Alcymene puellaris* (Reed 1920)	Phacopida	Calymenidae	Kionoceratid cephalopod	*Polygrammoceras bullatum* (Sowerby 1839)	Ludlow (Ludfordian)	Llangibby Beds, Wales	Molting	Davis et al. (2001, Fig. 8)
*Encrinuraspis beaumonti* (Barrande 1846)	Phacopida	Encrinuridae	Sphooceratid cephalopod	*Sphooceras truncatum* (Barrande 1860)	Ludlow (Ludfordian)	Kopanina Formation, Czech Republic	Molting	Barrande (1872, pl. 9, Figs. 24–26), Šnajdr (1990, p. 206–207), Kříž (1992, pl. 1, Fig. 18), Davis et al. (2001, Fig. 1)
Devonian								
*Phacops spedeni* Chatterton 1971	Phacopida	Phacopidae	Indeterminate nautiloid cephalopod	–	Early Devonian (Emsian)	Receptaculites Limestone, Taemas Formation, New South Wales, Australia	–	Noted in Chatterton et al. (2003, p. 166)
Scutelluids, phacopids, proetids, lichids, harpids	–	–	Rugose corals	–	Lower Devonian (Emsian)	Izarne Formation, Montagne Noire, France	Shelter during molting	Pedder and Feist (1998, p. 964)
*Phacops rana* (Green 1832)	Phacopida	Phacopidae	Atrypid brachiopod	*Pseudoatrypa* cf. *devoniana* (Webster 1921)	Middle Devonian, (Givetian)	Windom Shale Member, Moscow Formation, New York, USA	Protection from predators	Brett (1977, text-Fig. 1)
*Phacops* sp.	Phacopida	Phacopidae	Acleistoceratid cephalopod	*Acleistoceras* sp.	Middle Devonian, (Givetian)	Solon Member, Little Cedar Formation, Iowa, USA	Passive accumulation	Davis et al. (2001, Fig. 7)
*Cyrtosymbole* cf. *pusilla* (Gürich 1896)	Proetida	Proetidae	Clymeniid cephalopod	*Kalloclymenia* sp.	Late Devonian (Famennian)	Kowala Formation, Holy Cross Mountains, Poland	Shelter	Rakociński (2009, Fig. 5A-C)
*Omegops cornelius* Richter & Richter 1933	Proetida	Proetidae	Indeterminate nautiloid cephalopod	–	Late Devonian (Famennian)	Hongguleleng Formation, China	Molting	Zong et al. (2016, Fig. 2)
Indeterminate trilobite remains	*–*	*–*	Indeterminate nautiloid cephalopod	–	Late Devonian (Famennian)	Kowala Formation, Holy Cross Mountains, Poland	Shelter	Rakociński (2009, Fig. 5E)
Carboniferous								
*Cyrtoproetus* (*Cyrtoproetus*) *moravicus* (Přibyl 1950)	Proetida	Proetidae	Indeterminate ammonoid cephalopod	–	Mississippian	Formation unknown, Kulm Basin, Germany	Shelter for molting	Flick and Flick (2009, Figs. 2–5)

## Geological context

**Fig. 1:**
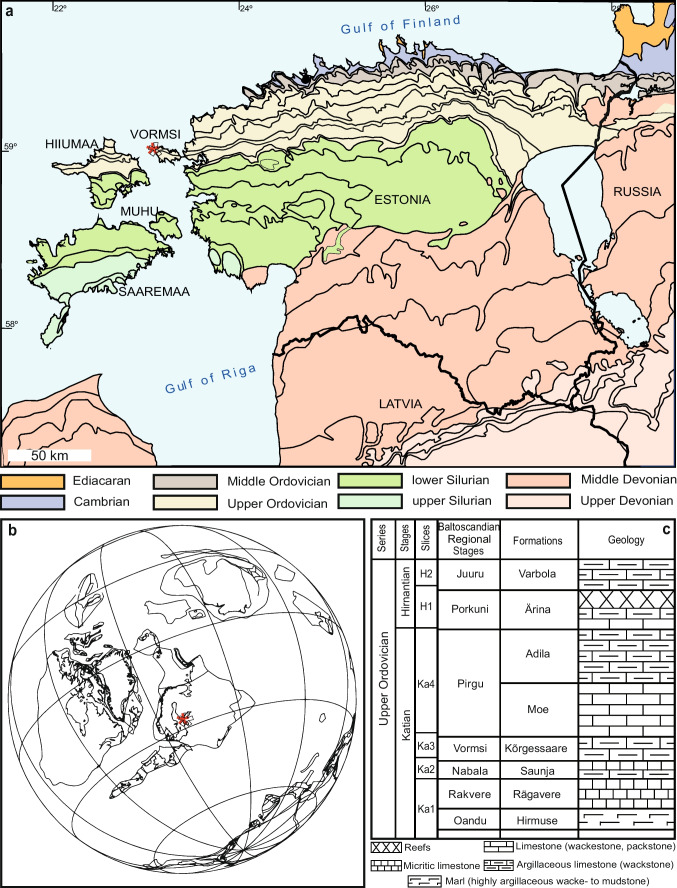
Geological, stratigraphic, and geographical information for specimen location. **a** Map showing local geology and specimen location (red star) in Estonia. **b** Paleogeography showing Baltica, 444 mya. Red star indicates the specimen location. Reconstruction constructed using BugPlates (Torsvik and Cocks 2009). **c** Stratigraphic section of local geology, showing position of the Kõrgessaare Formation

Vormsi is a low and flat island west of mainland Estonia that emerged from the Baltic Sea ~ 3000 years ago and rises to a maximum height of 11 m above sea level. Deposits below the thin Quaternary material are historically called the Lyckholm Layer and consist of the Upper Ordovician (middle to upper Katian shallow sea sediments mainly of bioclastic limestones that correlate upwards with the Nabala, Vormsi, and Pirgu regional stages; Fig. 1a; Schmidt 1858; Jaanusson 1944, 1956). The first detailed fossil collection on Saxby seashore, on the northwestern section of Vormsi, was by Sauramo (1929) who recorded a rich fauna over a 2-km-long section, proximal to the beach. A diverse assemblage of trilobites, brachiopods, cephalopods, gastropods, and corals totaling 46 taxa has been recorded. Furthermore, the fauna from the northern coastal region differs from the southern section. This was confirmed in Jaanusson (1944; 1956) who elected the substages based on this lateral faunal change—the older Kõrgessaare (in the northern region) and the younger Nõmmküla (in the southern region). This is due to a slight, 4–7° dip in the beds, which parallels the Paleozoic sedimentological belts observed in Estonia (Fig. 1). Despite this, both zones are considered part of the Kõrgessaare Formation, with the younger coral and calcitic vermiporellid algae-rich beds gradually transitioning into the overlying Moe Formation (see Kröger et al. 2017).

The Upper Ordovician Kõrgessaare Formation is a 10–20-m-thick limestone intercalated with marls and clay interlayers, becoming more argillaceous up-section. There is a ~ 1.7 m exposure along Saxby Beach at a cliff, but this is commonly covered by rubble and erosion. In the northern beach region, a discontinuity surface marks the upper Vormsi Stage (Einasto 2012). This is the most accessible area, as there is a road to the beach. This situation may have resulted in selective sampling bias in more recent collections, including our specimen. The fauna in the formation consists of brachiopods, gastropods, cephalopods, bryozoans, tabulate and rugose corals, and trilobites (Schmidt 1858, 1881; Sauramo 1929; Jaanusson 1944, 1956; Rõõmusoks 1998, 2000), mostly from the Vormsi Regional Stage. Documented trilobites include illaenids (*Parillaenus roemeri* (Volborth 1864) and *Parillaenus angustifrons* (Holm 1886)), proetids (e.g., *Ascetopeltis kertelensis* (Schmidt 1894), and *Cyphaspis* sp.), rare calymenids, enrinurids (*Erratencrinurus moe* (Männil 1958), *Erratencrinurus nebeni* Krueger 1971), pterygometopids (*Toxochasmops vormsiensis* Rõõmusoks 1998; *Valdariops angustus* Rõõmusoks 2000; *Valdariops eichwaldi* (Schmidt 1881)), lichids (*Amphilichas lineatus* (Angelin 1854), *Conolichas angustatus* (Beyrich 1846)), scutelluids (*Eobronteus laticauda* Wahlenberg 1818), harpetids (*Hibbertia* aff*. costatus* (Angelin 1854)), and asaphids (*Brachyaspis robustus* (Roemer 1861)). Except for the pterygometopids, all trilobite genera inhabited the deeper depositional environments of the Boda mud-mound (Suzuki et al. 2009) and during the Hirnantian trilobites with schizochroal eyes and better vision expanded into the pterygometopid niche (Hints et al. 2012; Ebbestad et al. 2015). The examined *T. vormsiensis* therefore likely lived relatively nearshore, in a warm, low-latitude epicontinental sea environment, rich in corals and shelly fauna.

## Methods and materials

The examined specimen is housed in the Geological collections of the University of Tartu (TUG) and assigned the specimen number TUG 1355–193. The specimen was photographed under LED lighting using an Olympus E-M1 MarkIII camera with a 12–45-mm lens. Images were stacked using OM Capture. Measurements of specimens were gathered using ImageJ (Schneider et al. 2012) and 3D Slicer 4.11 (Fedorov et al. 2012).

The specimen was micro-CT scanned on a GE PHOENIX v|tome|x scanner with a 240 kV X-ray tube at the Microscopy and Imaging Facility at the American Museum of Natural History (AMNH). The scan was run at 200 kV and 230 μA. Scan data were reconstructed using the GE software datos|x 2.1 and segmented in the software 3D Slicer 4.11 using the SlicerMorph toolkit (Rolfe et al. 2021). The reconstruction of the scan was exported as a.PLY file (Supplemental Document 1).

## Results

**Fig. 2:**
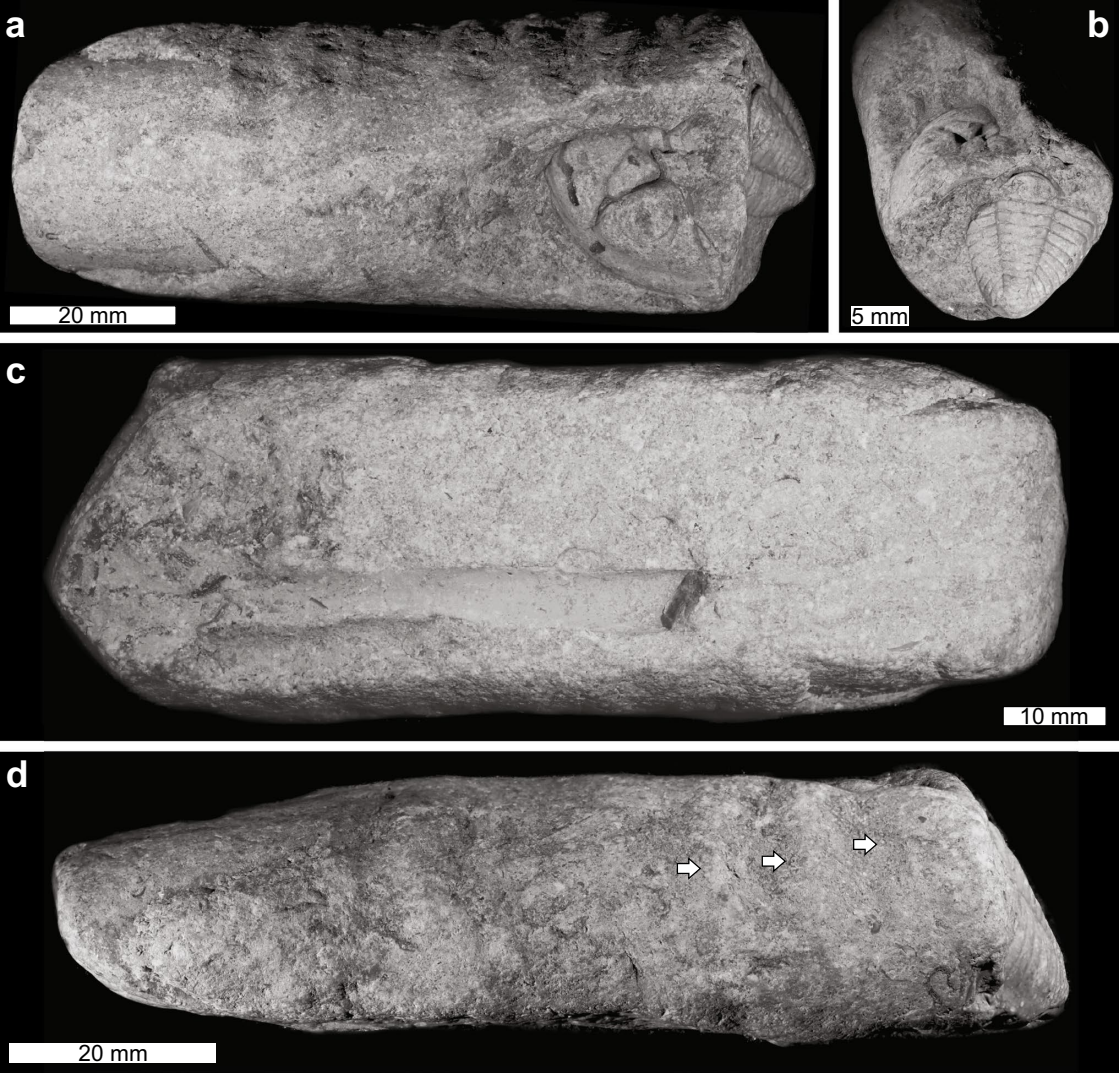
Examined *Toxochasmops vormsiensis* trilobite molt in *Gorbyoceras textumaraneum* nautiloid shell. TUG 1355–193. **a** View showing cephalon and trunk in oblique orientation. **b** View showing trunk with cephalon in oblique orientation. **c** View showing nautiloid siphuncle. **d** View showing external morphology and ornament (white arrows)

The specimen shows a molted exoskeleton of an individual of the trilobite *Toxochasmops vormsiensis* within the steinkern of a *Gorbyoceras textumaraneum* nautiloid (Fig. 2). The *G. textumaraneum* specimen measures 95.1 mm in length and 36.6 mm in width (across the aperture) tapering to 22.4 mm posteriorly. The trilobite is situated within the body chamber of the nautiloid. The chamber is entirely filled with sediment.

The *Toxochasmops vormsiensis* is partially preserved. The cephalon is disarticulated, missing the left eye (possibly due to erosion), and is partially covered by matrix on the right side; the cephalon sagittal length is 19.8 mm. The pygidium is disarticulated and partially covered by matrix on the left side; the pygidium sagittal length is 21.7 mm. The pygidial sagittal axis lies at an angle from the cephalic sagittal axis: ~ 10° to the right on a horizontal plane and ~ 60° declining posteriorly on a vertical plane. The cephalon and pygidium are separated by 11.6 mm, with the pygidium resting behind the cephalon in a telescoped configuration. The counterpart of a thoracic segment is located between the cephalon and pygidium. Upon examining the micro-CT scans, five to six articulated thoracic segments are identified in the matrix, under the cephalon.

## Discussion

Explanations for trilobites (Brett 1977; Davis et al. 2001; Chatterton et al. 2003; Fatka et al. 2009, 2021; Fatka and Budil 2014; Zong et al. 2016) and agnostids (Brongniart 1822; Suzuki and Bergström 1999; Chatterton et al. 2003; Fatka et al. 2009; Fatka and Kozak 2014) within larger animals have been summarized into four main behaviors: (1) molting in shelter, (2) hiding from predation, (3) shelter from sea floor disturbance, and (4) scavenging on food (Fatka and Kozak 2014). Although the exact reasons cannot be unambiguously presented here, these primary explanations can be explored to understand our specimen.

### Molting in shelter

Trilobites required quiet environments to complete ecdysis (Henningsmoen 1975; Brett 1977; Brandt 1993; Zong et al. 2016) and inhabiting the remains of other animals, such as empty cephalopod shells, would have been ideal for this purpose (Chatterton et al. 2003). The trilobite considered here is preserved within a molting configuration, demonstrating that the individual had entered the dead cephalopod conch to molt and excludes the possibility of the carcass having been washed into the nautiloid by passive fluid flow. While cephalopod shells may have been an ideal location for successful molting in paleoenvironments that lacked other safer areas to molt (Ladd 1929; Chatterton et al. 2003), the association of a trilobite within a nautiloid from the Kõrgessaare Formation is unique. This indicates that the environment likely had locations for successful molting, and, in this case, the trilobite was simply fortunate enough to locate a shell to molt within.

### Hiding from predation

Large, empty shells would have provided ideal shelter from predation, as well as molting (Brett 2003; Chatterton et al. 2003; Fatka and Kozak 2014). While there is no evidence for predation on the observed specimen, records of failed predation are known from Ordovician trilobites (see Owen 1985; Rudkin 1985; Zong 2021; Bicknell et al. 2022a, b; Fatka et al. 2022; Bicknell and Kimmig 2023). Although this specimen may not have been subject to predation, this interaction may indicate a preference for molting within cavities to avoid possible attacks (Brett 1990, 2003; Brett and Walker 2002; Fatka and Budil 2014; Fatka et al. 2021).

### Sea floor disturbance

Large shells represent ideal localities to survive periods of disturbances from storm events or rapid sediment inundation (Fatka and Kozak 2014). We are unable to determine if this situation can be excluded. As the paleoenvironment associated with this fossil is considered relatively nearshore, the trilobite may have entered the cephalopod to avoid disturbances and then may subsequently have molted.

### Scavenging on food

The cephalopod may have had decaying soft material ideal for benthic scavengers (Fatka and Kozak 2014) and would have attracted trilobites and other smaller animals to the shell (Fatka et al. 2009). There is no evidence for any other feeding activity preserved in the specimen. As such, we can exclude this option here as a primary justification for the trilobite entering the cephalopod.

Taken together, the evidence suggests that this interaction represents molting within a sheltered condition. However, this condition would also have allowed the trilobite to be protected from predators and from poor environmental conditions.

The molt configuration observed in this specimen—the pygidium wedged directly behind the cephalon in a telescoped manner—is common in *Toxochasmops* (McNamara and Rudkin 1984, figs. 1, 3; Rõõmusoks 1998, pl 1, fig. 8; pl 2, fig. 13). During exuviation, the cephalothoracic joint disarticulated, similar to Salter’s molting mode (Henningsmoen 1975). The cephalon was therefore molted in a way that allowed the trilobite to move anteriorly on an angle and wedge the pygidium against the cephalon, facilitating its removal (McNamara and Rudkin 1984; Budil and Bruthansová, 2005). Previously records of this molt configuration are of specimens in non-sheltered conditions (McNamara and Rudkin 1984). In these examples, few to no thoracic segments are observed, indicating the thorax is shed elsewhere (McNamara and Rudkin 1984). In our specimen, most of the thorax is preserved under the cephalon, as would be expected from molting in shelter. Further, TUG 1355–193 demonstrates that this complex exuviation technique was possible within enclosed spaces.

The limited space within the shell would have presented some complications, such as escaping from the shell after molting. Passive sediment in-filling of an open structure on the sea floor likely resulted in a partly filled shell when the trilobite entered to molt (Hewitt 1988). This would have further limited the space for movement. Given the molt size, the trilobite likely filled up over half of the remaining space and would have had to move farther into the conch after molting. If the individual did leave the shell after molting, it likely rotated itself and moved over its molt. Such flexibility would have been possible in its soft-shelled condition (Drage et al. 2019) as the trilobite may have been more dorso-ventrally compressible, permitting movement through the limited space (Drage and Daley 2016). This exit from the conch could have taken minutes to days (Zong et al. 2016). Alternatively, the individual may have been trapped behind its molt, unable to escape. The Kõrgessaare Formation does not preserve soft-bodied fossils and, as such, we cannot test this possibility. Due to this limitation of the fossil record, we present both possible outcomes here.

By considering the history of trilobite molting within larger organisms, we can explore when in time and where in the group this cryptic behavior arose (Table 1). Although it was more common within agnostids (see Chatterton et al. 2003; Valent et al. 2008; Fatka et al. 2009; Fatka and Kozak 2014), hiding during molting had an origin in Cambrian agraulid trilobites (Valent et al. 2008). There is an increase in the prevalence of these interactions in the Ordovician asaphids, calymenids, harpetids, odontopleurids, and pliomerids (Table 1). This increase may reflect a biological signal, or it may be sampling bias. Additional examination of trilobite interactions during the Cambrian would help resolve this question. The long record of trilobites concealing themselves within larger animals may also reflect the rise of larger, more effective durophagous predators that targeted smaller trilobites (Brett 1990; 2003; Bicknell and Paterson 2018). Additionally, the increased abundance of this behavior likely gave trilobite species a protective advantage, adding to their success.

Habitation of animals in cephalopod shells is also observed in the Mesozoic (Fraaye and Jäger 1995a, b; Davis et al. 2001; Fraaije and Pennings 2006; Vullo et al. 2009; Klompmaker and Fraaije 2012; Landman et al. 2014; Nyborg et al. 2014; Smith and Holland 2016; Fraaije et al. 2020; Bicknell et al. 2021). The apparent increase in the diversity of animals showing this association reflects the use of cavities as a means of shelter, a food source, or for reproduction (Fraaye and Jäger 1995a, b; Nyborg et al. 2014). Despite the Mesozoic record, there are large gaps in information regarding these behaviors and associations, especially in the later Paleozoic trilobites (Table 1). We therefore propose the ongoing examination of cephalopod shells with the aim of documenting the use of these remains by benthic animals across the Phanerozoic.

### Supplementary information

Below is the link to the electronic supplementary material.

Supplementary file1 (PLY 36277 KB)
